# Domestic Correspondence

**Published:** 1872-04-15

**Authors:** Ed. J. Cleveland

**Affiliations:** Lawrence, Mich.


### Domestic Correspondence.

Lawrence, Mich., Feb. 7th, 1872.

Prof. Andrews — *Dear Doctor:* In the summer of 1868 I removed an epithelial cancer from the back of the hand, extending obliquely from near the first joint of the forefinger to the tendon of the little finger, at the metacarpal articulation, oval in shape, like a great wart, and covered by a scab. It had been previously "burned" out by a cancer quack at Paw Paw, twice.

The wound healed well; but in the fall another wart appeared between the metacarpal bones of the thumb and fore-finger. I was in Chicago at the time, and found on my return in the spring a cancer fully as large as the previous one, ulcerated and discharging a very fetid sero-purulent fluid. The patient was an old man eighty-two years old, but vigorous, and as young as most men at sixty-five.

There were two other warts forming upon his face, looking more like crusts than anything else. I found it impossible to correct the fetor; and in spite of iron and tonics he grew cachectic. The ulcer began to bleed. Seeing his almost hopeless condition, and being unable to bear the horrible odor emanating from the ulcer, he determined to submit to an amputation, which, under the circumstances, I deemed justifiable, though, owing to the manifest cachexy, almost hopeless. I thought, on the whole, that he would live longer after an operation than without it. In June, 1869, I amputated in the middle third of the arm. He bore the operation extraordinarily well, and convalesced more rapidly than any one I ever saw. I removed at the same time the warts on his face. Put him on iodide of iron; and he made for himself a pill of the dried juice of the phytolacca decandra, and also drank an infusion of the bark of the red alder, having read that these were good for cancer. The cachexy disappeared; and although much broken down and unable to work, he enjoyed good health for over two years, and died last fall of pneumonia. This is the only case of cancer that I have cured.

I have two cases of cancer of the breast under treatment — one for two years, with galium — and the tumor in one case has almost entirely disappeared, and the other has diminished to one-third its former size. I tried arsenic and conium in the latter case with no effect. I was so much impressed with the old man's tea of tag alder, that I used it in both of these cases, and noticed that iron seemed to do five times as much good while they took the infusion as it did without. Whether galium is a specific for cancer or not, I can say that it diminishes the pain better than anything I have used or seen used.

I have not tried the cundurango yet, but mean to compare its effect with that witnessed in these cases. Cancer is hereditary with both these women. The cancer in the first case was recent and growing, though it had not attained a large size, being irregular and not more than an inch in its largest diameter. A curious fact was noticed in the family history, that most of the males had died of tuberculosis, while the females died of cancer. She has two aunts in Ohio who are quite old, in whom the disease reached a certain point, and has been dormant for years.

In the second case, the parents are cousins. I could learn positively of only one death in the family from cancer. This was an aunt on the mother's side who had been operated on, and the disease had returned. They thought that a cousin had died of the disease, but I could not learn positively. The tumor in the second case was irregularly oval, nodulated, extending downward from the nipple, which was not retracted; was about three inches at the longest diameter; was of nine years standing, though it had not grown any for six years; axillary glands somewhat enlarged; lancinating pains very severe. When I first saw the patient she was sallow and cachectic; but from the effect of the iron I gave her, and her history, I am satisfied that this appearance was due to malarial influences, and that the constitution was not affected.

Knowing how interested you are in everything pertaining to our profession, I thought I would send you these sketches of two of my *odd* cases. I think I am favored with a great many odd ones. I remain,

Yours truly,

ED. J. CLEVELAND.

